# Who may benefit most from future vitamin D intervention trials: do not forget patients on continuous renal replacement therapy

**DOI:** 10.1186/s13054-020-02910-w

**Published:** 2020-04-28

**Authors:** Patrick M. Honore, Aude Mugisha, Luc Kugener, Sebastien Redant, Rachid Attou, Andrea Gallerani, David De Bels

**Affiliations:** ICU Department, Centre Hospitalier Universitaire Brugmann-Brugmann University Hospital, Place Van Gehuchtenplein, 4, 1020 Brussels, Belgium

We read with great interest the recent paper by Martucci et al. who concluded that high-dose vitamin D3 supplementation was associated with a reduction in 28-day mortality in a mixed population of critically ill adults with vitamin D deficiency [1]. Their analysis also attempted to identify who may benefit the most from future vitamin D intervention trials [1]. We would like to make some comments. In their paper, Martucci et al. did not include data regarding patients with acute kidney injury (AKI) needing renal replacement therapy (RRT) within the critically ill population they studied [1].

25-Hydroxyvitamin D has a molecular weight of 400 Da and thus should be easily removed by dialysis [2]. However, most of 25-hydroxyvitamin D is bound to vitamin D-binding protein, which has a molecular weight of 10 kDa and needs convection to be removed [3]. Convection, the main modality used in continuous renal replacement therapy (CRRT), can drastically reduce levels of both 25-hydroxyvitamin D and vitamin D-binding protein [3, 4]. Uhlin et al. found that the use of convection was very deleterious for 25-hydroxyvitamin D levels, with a significant reduction in 25-hydroxyvitamin D levels following the switch to online hemodiafiltration [5]. We agree with the authors that critically ill patients would benefit from vitamin D intervention trials, and given the significant loss of vitamin D in patients undergoing RRT, we believe this group of patients in particular should be a focus of further study. Studying these patients is made complicated by technique-related differences (type and frequency of RRT, type of membrane used, etc.) and individual patient pharmacokinetic variations (changes in volume of distribution, degree of protein binding, residual renal function, intestinal absorption of vitamin D, etc.). Perhaps the most pragmatic approach in a future study would be to give a loading dose of vitamin D, similar to that of the VITdAL-ICU study [1], and then monitor blood concentrations, like we do for antimicrobials, to guide further dosing requirements.

## Authors’ response

Gennaro Martucci, Dayre McNally, Dhruv Parekh, Karin Amrein

Dear Editor,

We read with great interest the letter by Honore and collaborators on the supplementation of vitamin D in critically ill patients undergoing continuous renal replacement therapy (CRRT). We agree with the approach suggested for several reasons: First, from the methodological point of view, the accumulation of adequate data on basic science, including observational data on pharmacodynamics and pharmacokinetics applied in clinical practice, should be mandatory to better target the population that would benefit more from a specific intervention tested in an RCT [6–8]. Second, from the clinical side, this observation is highly relevant, since in our post hoc analysis chronic kidney disease was associated with a reduction of the survival benefit in the treatment group.

Unfortunately, we are unable to provide the requested data as the VITDAL-ICU Trial recruited from 2010 to 2012, and though patients on dialysis were not excluded, they were only a small minority of the study population. Also, at the time of the trial, CRRT was not as frequently used as today, and data on CRRT were not recorded systematically [9].

We would like to stress that clinicians administering vitamin D should be aware of the factors that may contribute to acute changes in vitamin D levels [10]. Both for changes in volume distribution and for cartridge absorption and filtration, the level of vitamin D is lower in critically ill patients on CRRT, and the same can be hypothesized in the case of supplementation [11]. In fact, it is clear that CRRT is able to reduce the plasma level of proteins, foremost albumin, but also vitamin D-binding protein with its ligand vitamin D [12]. This fact alone should be reason enough to prompt higher doses of vitamin D supplementation to be effective, though there are no data to support this hypothesis.

Indeed, moving forward from the VITDAL study and ideally continuing it, the VITDALIZE trial, a European multicenter RCT focused on vitamin D supplementation in patients with severe vitamin D deficiency, will also consider patients with ongoing CRRT, and will likely give an answer to both questions: variation of vitamin D plasma levels during CRRT, and the ability of high doses of cholecalciferol in reducing mortality [13].

## Data Availability

Not applicable.

## Abbreviations

AKI: Acute kidney injury
RRT: Renal replacement therapy
CRRT: Continuous renal replacement therapy
